# Impact of Y chromosome AZFc subdeletion shows lower risk of fertility impairment in Siddi tribal men, Western Ghats, India

**DOI:** 10.1186/s12610-014-0017-5

**Published:** 2015-01-22

**Authors:** Shivaprasad Holenarasipura Sathyanarayana, Suttur Srikanta Naik Malini

**Affiliations:** Molecular Reproductive and Human Genetics Laboratory, Department of Studies in Zoology, University of Mysore, Manasagangothri, Mysore, 570006 Karnataka India

**Keywords:** Male infertility, Y chromosome, Subdeletions, Siddi tribe, Ethnicity

## Abstract

**Background:**

India is characterized by the presence of a large number of endogamous castes, tribes and religions, having second largest concentration of tribal population in the World with differed genetic ethnicity, lifestyle and environmental habitat from those of mainstream population. Lack of data is constraint when it comes to tracking the tribal population health status, specifically reproductive health aspects by experimental approaches. The male fertility impairment depends on Y chromosome azoospermia factor c (AZFc) subdeletions, which varies highly in different geographical populations and in an Indian admixed population the frequency and effect of deletion on fertility is relatively poorly documented. Therefore, the current study has been initiated to enumerate and characterize the strength of association between Yq11 AZFc subdeletions and fertility impairment among Siddi tribal men of Western Ghats, India.

**Methods:**

Here, using predesigned performa we collected personal as well as familial information of 200 volunteered male subjects and grouped them into: (i) 104 married individuals with proven fertility, and (ii) 96 unmarried men with unknown fertility status. Quantification of reproductive hormones such as follicle stimulating hormone (FSH), leutinizing hormone (LH) and testosterone were studied. Oxidative stress markers like total antioxidant capacity (TAC) and super oxide dismutase (SOD) along with analysis of five sequence tagged site (STS) hotspot markers were employed for mapping of Y chromosome AZFc subdeletions. Statistical analyses were performed using SPSS software.

**Results:**

Hormonal analysis and estimation of oxidative stress markers showed normal values with no significant differences between two subgroups. However, the Y chromosome AZFc subdeletion mapping revealed evident results as an individual displayed absence of STS sY1191 marker indicating b2/b3 deletion, whereas rest of the subjects exhibited no deletion for all the five STS markers. While, the individual has fathered two children, at this point it is difficult to draw a causal link between the observed deletion and its effect on fertility.

**Conclusion:**

Thus, our current study suggests that the association between AZFc subdeletions with its effect on infertility varies highly in this study cohort compared to other Indian ethnic groups, exhibiting lower risk factor and non-association reaching insignificance among Siddi tribal men.

**Electronic supplementary material:**

The online version of this article (doi:10.1186/s12610-014-0017-5) contains supplementary material, which is available to authorized users.

## Background

The human Y chromosome plays a vital role in fertility factor comprises of several male-specific gene families, which are exclusively expressed in the testis [1]. Specifically, the Yq11 segment consisting of three azoospermia factor (AZF) encoding regions namely, AZFa, AZFb and AZFc that accounts for structural abnormalities in the male-specific region of Y chromosome (MSY) [2]. While, the AZFc region is more prone to *de novo* mutations than AZFa and AZFb regions, the emerging findings of AZFc functionality and polymorphism in spermatogenesis has gained renewed scientific interest in the recent years [3,4]. AZFc complete deletions are associated with hypo-spermatogenesis condition causing abnormal and decrease sperm production, whereas the AZFc subdeletions in few copies of eight gene families results in spermatogenic impairment [5]. However, several studies have reported the occurrence of these subdeletions in fertile individuals as well, with varying genotypic and phenotypic effects [6,7].

**Figure 1:**
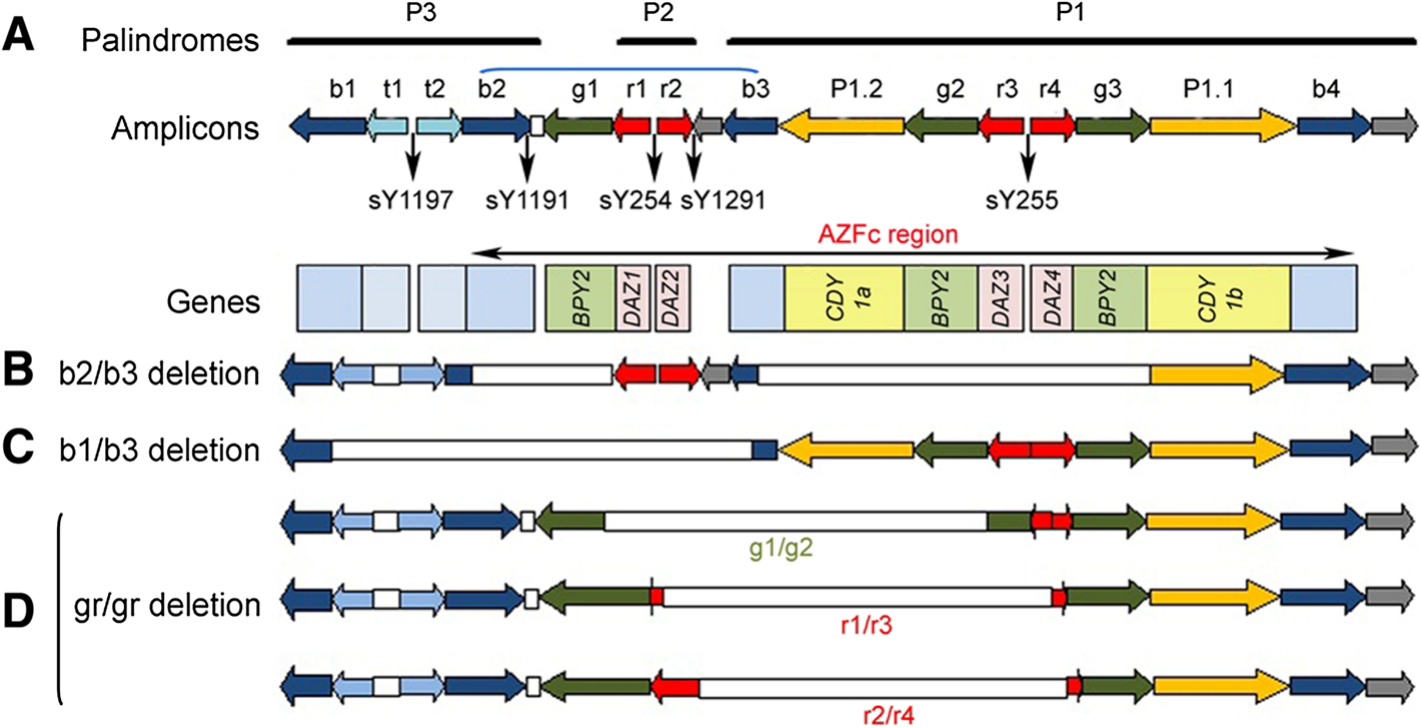
**Map of the AZFc region.** The AZFc subdeletions pattern in Y chromosome: **(A)** Schematic illustrating the palindromes and amplicons in the AZFc region. Locations of different STS markers that are employed to screen the subdeletions are indicated below the ampliconic bar, where the colour boxes depict the protein encoding genes in the AZFc region (gene names are presented inside the colour box). The AZFc subdeletion patterns for **(B)** b2/b3 deletion (blue arch in **(A)** indicates the b2/b3 deletion due to homologous recombination between the amplicons), **(C)** b1/b3 subdeletion and **(D)** gr/gr deletion with g1/g2, r1/r3 and r2/r4 that removes different set of genes are indicated with open bar.

In general, the AZFc region encompasses of eight gene families including *BPY2, CDY, DAZ, CSPG4LY, GOLGAZLY, TTY3.1, TTY4.1*, and *TTY7.1*, of which, first five genes encodes for proteins that are essential for spermatogenesis (Figure 1A). Interestingly, *BPY2, CDY* and *DAZ* belongs to multicopy gene family that respectively comprise of 3 copies of the *BPY2*, 2 copies of the *CDY1*, and 4 copies of the *DAZ* [8]. Recently, different types of AZFc subdeletions have been identified such as, gr/gr, b1/b3 and b2/b3 deletions, but their effect on spermatogenic impairment is still remains unclear. Among these, gr/gr subdeletions are frequently reported with prevalence ranging from 2.1% to 12.5% in the infertile individuals and 0% to 10.2% in the fertile men [9]. Essentially, these are the group of different deletions caused by recombination flanking g1/g2, r1/r3 and r2/r4 amplicons in P1 and P2 palindromes (Figure 1D) and accounts for reduction in half of the AZFc region that causes loss of *DAZ, CDY1* and *BPY2* gene copies in combination, which is anticipated as a risk factor for decreased sperm count [8,10-12]. Several independent studies from Dutch, Spanish, Italian, Australian, Portuguese and Han Chinese population reported 0% to 5.3% association of gr/gr deletion with spermatogenic disruption in controls and 3.2% to 10.6% of correlation with male infertility among infertile individuals. On the contrary, studies from French, German, Srilankan, Han Chinese, Brazilian, Japanese, Chilean and Moroccan population accounted 1.8% to 33.9% of non association of gr/gr deletion with spermatogenic impairment and 2.1% to 23.9% among infertile subjects [9,13]. In contrast, despite losing most of the AZFc gene copies (1.8 Mb deletion), the b2/b3 subdeletion appears to be polymorphic without any obvious effect on fertility. Although, the deletion frequency of b2/b3 is less common compared to gr/gr subdeletions and its association with infertility are documented in Chinese population but not in European population, the effect of b2/b3 deletion on spermatogenesis remains to be elusive [14-18]. Considering that b2/b3 deletions are fixed in haplogroup N of European population with no obvious effect on fertility. These results illustrates that the incidence of b2/b3 deletions outside of haplogroup N may represent a risk factor for spermatogenic impairment [19].

Despite several studies have reported the risk factors for the above mentioned deletions, the precise nature as well as the size of these deletions with possible impact on spermatogenesis and fertility impairment across different ethnic groups are not clearly understood [20]. For instance, in India the Y microdeletion studies are largely restricted to clinical samples, which include infertile and/or subfertile patients with wide variation in deletion frequency (0% to 28%) [21-27]. The strong basis for such large difference could be the dissimilar sample size and the ethnic background. Additionally, uniformity in employing same number and type of STS markers for deletion assessment and in some instance usage of very limited or non EAA/EQMN (European Academy of Andrology and European Molecular Genetics Quality Network) markers may contribute for variations in deletion frequency [28-31]. However, the positive association between the Y chromosome AZFc subdeletion and infertility condition are reported in two independent studies from North and South Indian clinical samples [15,32]. Given that Indian population with various ethnic backgrounds offers an excellent study cohort to examine the impact of Y subdeletion on infertility, the current study has been initiated to examine and analyze the AZFc subdeletion among Siddi tribal men as their genetic background, lifestyle, food habits, the geographic location and influence of environmental factors are different from urbanized population of South India.

**Figure 2:**
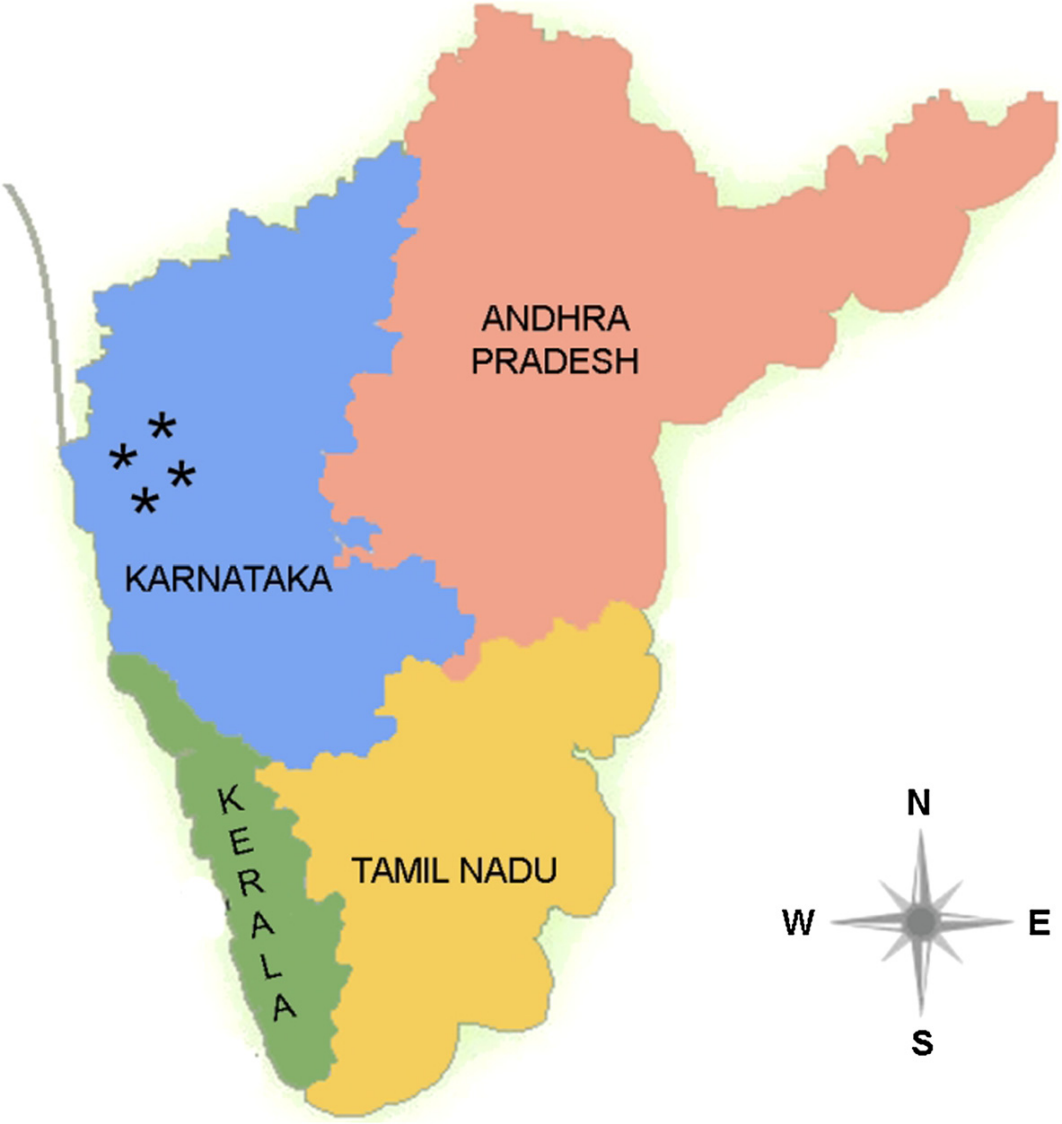
**Geographic map of South India: Map shows the Southern region of India.** Star mark represents the geographic location of Siddi tribal population in Western part of Karnataka.

Siddi’s are a distinctive tribal community with an African ancestry, settled majorly in Karnataka and scantily in some parts of Gujarat as well as in Andhra Pradesh, with total population of 0.25 to 0.30 million [33]. Around 1100 AD, the existence of this tribal group in Western India has been first documented and later, in 13th century Nawabs and Sultans of India imported large group of Siddi individuals from Africa as their slaves [34]. Interestingly, more recent genetic studies provide evidences that the Siddi’s are not only descents from Africans, but they also seem to have a European and South Asian ancestry [26,35,36]. Currently, this tribal group is largely settled in and around Western Ghats forest of Karnataka (Figure 2) with agriculture and daily wage as their primary occupation. More importantly, as Siddi’s migrate less often compared to other tribal groups and out breeding is not practiced in this community, allowed us to reach them easily considering their lineage more conserved and unique from rest of the admixed Indian population.

As stated before in the context of Indian literature, till date the correlation between the male infertility and reproductive functional status using the molecular approaches are predominantly restricted to clinical population and interestingly, there are no conspicuous data available in any tribal population. Here, we have explicitly focused on AZFc euchromatic region of the Y chromosome to investigate and associate the functional role of AZFc in an individual reproductive fitness. For this purpose, we have recruited the married and unmarried male subjects from Siddi tribal population, Karnataka, India. Most importantly, to best of our knowledge this is the first original report from Karnataka, which screened the AZFc subdeletion in Siddi tribal men and in addition, we believe that our current findings may also provide an interesting insights about the African population as well.

## Methods

### Ethical statement

Current study was approved by the Institutional Human Ethical Committee (IHEC), University of Mysore, Mysore, India (IHEC-UOM/50/2011-12). Before commencement of this study, the written informed consent was obtained from all the study participants. Additionally, all the subjects were briefed about the study as well as the sample collection and its usage.

### Study population

The study was carried out in different places of Siddi tribal settlement situated close to Western Ghats forest in and around Uttar Kannada district, Karnataka, South India (Figure 2). Our study cohort consisted of 200 volunteer Siddi tribal men with an age group ranging from 18 – 45 years. The predesigned proforma was prepared to collect information about the age, health status, food habits, marital status as well as reproductive history in case of married individuals, and other relevant information required for this study. The main exclusion criteria for the current study include men who were above 45 years of age and the subjects who had history of contagious infections, allergies or any other sort of health issues. The study subjects were grouped into: (i) Group 1 include 104 healthy Siddi tribal men who were married and fathered one or more children with proven fertility without any medical aid. (ii) Group 2 comprises of 96 Siddi men who were unmarried with unknown fertility status.

### Hormonal analysis

Serum hormone levels for FSH, LH and testosterone were analysed in 200 male subjects and determined in duplicate reaction using commercial available enzyme linked immunosorbent assay kit (Labor Diagnostika Nord GmbH, Germany) as per the manufacturer’s protocol. The optical density (OD) was measured using flash multimode reader (Thermo Varioskan, USA) and the obtained values were compared and analyzed with respect to standard reference ranges of serum FSH (1.4 – 14 mIU/mL), LH (1.2-9 mIU/mL) and testosterone (3.0 – 12.0 ng/mL) provided in the kit.

### Quantification of TAC and SOD

The TAC estimation was carried out in duplicates using 5% trichloroacetic acid solution (TCA) and vitamin-C as standard as described by Prieto et al. [37]. The SOD estimation was carried out using previously described protocol by Kazari et al. [38] with minor modification.

### Deletion analysis of *DAZ* gene cluster and AZFc subdeletion mapping

Genomic DNA was extracted from the peripheral blood samples using QIAmp DNA Blood Mini Kit (Qiagen, Germany) as per the manufacturer’s protocol. Standard PCR reactions were carried out according to the (EAA/EMQN) guidelines [31]. We tested all the 200 samples for AZFc *DAZ* gene cluster deletion using STS markers - sY254, and sY255. In addition, we also screened for the presence and absence of AZFc subdeletion using specific STS markers [11] sY1291 for gr/gr deletion, sY1191 for b2/b3 deletion and sY1197 with sY1291 for b1/b3 deletion in all the samples (Additional file 1: Table S1).

The reaction conditions for all the five markers were initial denaturation 94°C for 3 minutes and then, 30 cycles at denaturation 94°C for 30 second, annealing 58°C for 30 second (for sY254, sY255 and sY1197) and 60°C for 30 second (for sY1291 and sY1191) and extension 72°C for 30 second with final elongation at 72°C for 5 minutes. All the reactions were performed using male positive control, female negative control and water as blank. Samples in which the deletions were detected in first round were reconfirmed by repeating the PCR analysis at least twice. The reaction products were analysed by means of 2% agarose gel. The *DAZ* gene cluster deletion was observed by the absence of corresponding bands for the markers sY254 and sY255. The gr/gr deletion was screened by the absence of marker sY1291, whereas b2/b3 deletion was detected by sY1191 and finally, the absence of sY1197 with sY1291 indicates b1/b3 deletion in the subjects.

### Statistical analysis

The data was analyzed using the statistical software SPSS version 21. The mean and standard deviation values were calculated for the continuous variables. Independent *t*-test was performed for hormone analysis, TAC and SOD within the categorized age groups among married and unmarried cohort (18 to 30 years, 31 to 40 years and 40 years above), where *p* value ≤ 0.05 was considered as statistically significant after the test.

## Results

In the present study, group one includes 104 married males with an average age of 29.67 ± 9.69 years with proven fertility and an average of 2.61 ± 0.95 children per individual. The second group consists of 96 unmarried males with unknown fertility status and an average age of 29.04 ± 9.68 years (Additional file 2: Table S2). The predesigned proforma used to collect the detailed information of all the subjects revealed that individuals in both the group consume healthy, nutritious diet with vegetarian and non-vegetarian as their major food source. Furthermore, compared to unmarried group, married individuals showed slightly higher response for alcohol consumption. In contrast, smoking and tobacco usage in both married and unmarried group is on higher side. To our surprise, there are no other practices in this tribal community that possibly affect their health condition and lifestyle factors.

Interestingly, in hormonal analysis except the slight variation in LH levels, we have not observed any statistical significant changes in FSH and testosterone levels among married and unmarried individuals across different age groups. All the married individual showed normal range with group mean values for LH 8.14 ± 3.45 mIU/mL, FSH 3.03 ± 0.85 mIU/mL and testosterone 9.37 ± 8.20 ng/mL, whereas unmarried group showed the average range for serum LH 7.48 ± 3.1 mIU/mL, FSH 3.00 ± 1.05 mIU/mL and testosterone 8.79 ± 6.93 ng/mL, in comparison with the standard reference values (Table 1). However, independent *t*-test analysis for multiple comparisons for serum hormonal analysis of LH within different age groups among married and unmarried individuals showed a significant difference in 18–30 years (*p* ≤ 0.004), and 40 years and above age group (*p* ≤ 0.009), but not in 31–40 years of age group (*p* = 0.703) (Table 1). The influence of altered LH levels among this group may be due to varied sample size employed for multiple comparison analysis.

**Table 1:** **Statistical analysis of serum hormonal analysis: mean and standard deviation values for serum hormonal analysis among married and unmarried individuals of different age groups**

**Sl. no.**	**Age group**	**Hormone**	**Marital status**	**Mean ± SD**	**F**	***p*** **value**
1	18-30 years	LH	Married	8.85 ± 3.66	10.05	0.004*
			Unmarried	7.16 ± 2.93		
		FSH	Married	2.95 ± 0.70	5.74	0.706
			Unmarried	3.01 ± 1.03		
		Testesterone	Married	9.01 ± 8.19	2.90	0.711
			Unmarried	8.52 ± 6.65		
2	31-40 years	LH	Married	7.78 ± 3.40	1.90	0.703
			Unmarried	7.24 ± 1.13		
		FSH	Married	3.20 ± 1.11	0.39	0.742
			Unmarried	3.37 ± 1.15		
		Testesterone	Married	10.63 ± 8.90	3.76	0.659
			Unmarried	8.94 ± 5.75		
3	41- 45 years	LH	Married	7.13 ± 2.80	10.32	0.009*
			Unmarried	11.03 ± 4.71		
		FSH	Married	2.96 ± 0.64	3.78	0.0375
			Unmarried	2.67 ± 1.07		
		Testesterone	Married	8.21 ± 7.15	2.78	0.0378
			Unmarried	11.21 ± 10.5		

*p*-values are calculated using independent *t*-test for multiple comparisons.

*p < 0.05 indicates significant difference.

Similar to serum hormonal analysis, we have not observed any changes in serum TAC levels in married and unmarried individuals, who showed the values with an average of 135 ± 29.05 mM and 130.21 ± 28.01 mM, respectively (Table 2). Additionally, the observed SOD values for married (0.84 ± 0.30 units of SOD/mg of protein) and unmarried individuals (0.92 ± 0.27 units of SOD/mg of protein) showed statistically insignificance (Table 2). Taken together, the statistical significance values determined using the independent *t*-test between two groups for TAC and SOD parameters indicates that the study cohort may have very less possibility of infertility risk factors associated with oxidative stress.

**Table 2:** **Statistical analysis of TAC and SOD assay: summary of mean and standard deviations values for estimation of TAC and SOD with independent sample** ***t-*** **test applied for multiple comparisons for continues variables among married and unmarried age groups**

**Sl. no.**	**Age group**	**Test for**	**Marital status**	**Mean ± SD**	**F**	***p*** **value**
1	18-30 years	TAC	Married	127 ± 22.49	4.54	0.696
			Unmarried	128.90 ± 28.66		
		SOD	Married	0.82 ± 0.31	0.76	0.109
			Unmarried	0.91 ± 0.27		
2	31-40 years	TAC	Married	147 ± 34.23	0.91	0.901
			Unmarried	146.11 ± 18.04		
		SOD	Married	0.87 ± 0.31	0.21	0.280
			Unmarried	0.72 ± 0.24		
3	41- 45 years	TAC	Married	136.93 ± 29.54	0.03	0.282
			Unmarried	123.94 ± 25.74		
		SOD	Married	0.90 ± 0.27	12.51	0.511
			Unmarried	0.97 ± 0.10		

*p < 0.05 indicates significant difference.

**Figure 3:**
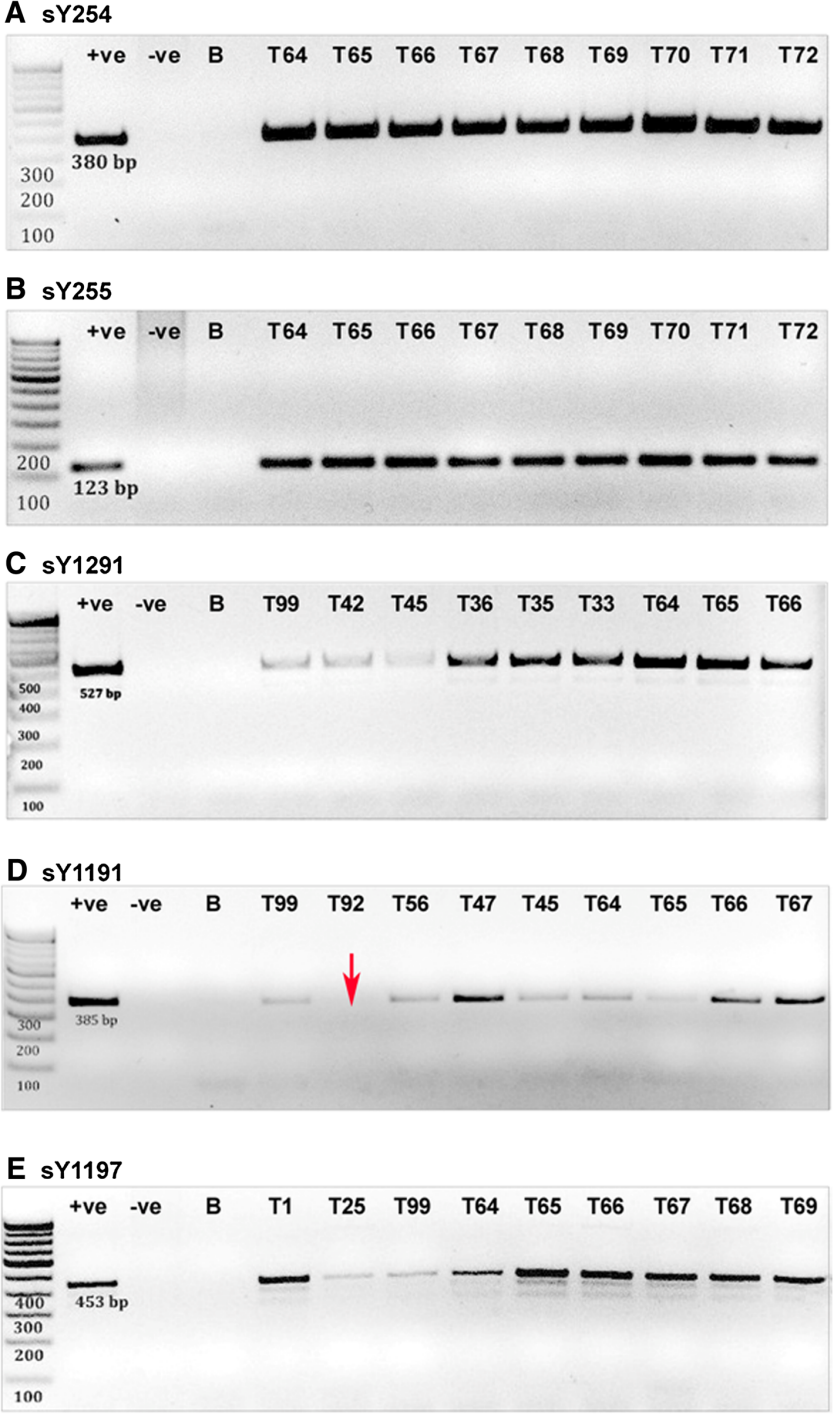
**Image of agarose gel electrophoresis: (A to E) Gel image showing PCR products for various STS markers that are used to screen the Y chromosome AZFc subdeletions in Siddi tribal men.** Red arrow **(D)** indicates the deletion of sY1191 marker in sample number T92. Lane 1 represents 100 bp marker. +ve, − ve and B corresponds to control genomic sample, female genomic sample and water blank respectively.

Although the predesigned proforma and biochemical analysis rule out any fertility related issues in this group, the genetic variations that may possibly contribute for genotypic and phenotypic alterations in the male-specific genes are still unclear. Surprisingly, AZFc subdeletion mapping revealed the absence of deletions for all 5 specific STS markers (Additional file 1: Table S1) that are tested in unmarried individuals (n = 96) (Additional file 3: Table S3). However, none of the study subjects in the married group (n = 104) showed AZFc subdeletion for sY254, sY255, sY1291 and sY1197 (Figure 3A, B, C, E). In contrast, only one person (0.50%) out of total 200 individuals showed AZFc subdeletion for sY1191 STS marker, which indicates the b2/b3 deletion due to homologous recombination between the b2 and b3 amplicons in the AZFc region (Figure 3D, lane T92). When we cross examined the individual personal information, we found that he was married and has fathered two male children, suggesting that the b2/b3 deletion seems to have no impact on fertility impairment.

## Discussion

In the current study, we have examined a total of 200 Siddi males for biochemical assays and also, conducted STS based PCR analysis for AZFc subdeletion mapping to understand the functional status of Y chromosome and its impact on fertility. Interestingly, we have not observed any clear association between changes in the hormonal and oxidative stress levels with AZFc subdeletions and their effect on reproductive status of Siddi tribal men. Thus, by reporting the negative co-relationship of AZFc subdeletion and fertility impairment, the current study has made a significant contribution in understanding the frequency of AZFc deletion and its non-deleterious effect on fertility among Siddi men.

In this context, it is noteworthy to mention that most reported studies in Indian tribal population are predominantly restricted to reproductive health care and fertility related issues in women, whereas male-specific reproductive literature are limited. For instance, effects of inbreeding on fertility and sterility among urban and rural population of South India has been evaluated by assessing the type of consanguineous marriage, marital age, duration of marriage, number of pregnancies, live births and child mortality [39]. Kumar et al. [40] conducted a factorial experiment to determine the outcome of sex and survivorship to fertility in Kamara tribes from Chhattisgarh [40]. To quote an another example, age of menarche, marriage and menopause along with nutritional status of mother during prenatal and post pregnancy periods are well documented in Dhur and Gond tribal women of Chhattisgarh [41]. Taken together, to best of our knowledge for the first time in South India, we have systematically screened 200 Siddi tribal men and analysed their genomic samples for the presence and absence of Y chromosome AZFc subdeletions.

Furthermore, several studies have implicated the clinical significance of oxidative stress by demonstrating the causal link between oxidative damage and occurrence of male infertility, thereby suggesting infertile men are more likely to have reduced levels of TAC compared to fertile individuals [42]. In addition, cigarette smoking and alcohol consumption can increase the oxidative stress levels that in turn affect the individual’s fertility [43,44]. Despite our study subjects exhibited higher response for smoking and alcohol consumption, due to intake of nutrient diet that are rich in antioxidants, the subjects are relatively less prone to oxidative stress as evident from TAC and SOD analysis.

Earlier studies suggest that the spermatogenesis in males is initiated and regulated by the action of FSH and LH, and any changes in these hormonal levels may lead to abnormal sperm profile with lower sperm count causing fertility impairment [45]. LH acts on Leydig cells in the testis and produces testosterone by enhancing the steroidogenesis [46]. The normal range of testosterone is essential for the development and proper functioning of the male accessory reproductive gland and impairment in hormonal levels may results in poor semen quality affecting individual’s fertility [47]. However, in our study the serum hormone estimation for LH, FSH and testosterone showed normal range among married and unmarried groups.

As stated earlier, the current study is mainly focused on the Y chromosome AZFc subdeletions that reduces the overall dosage between 1.6 – 1.8 Mb of the AZFc genes (*DAZ, CDY* and *BPY* copies) and global studies suggests that these deletions results in infertility, which vary according to ethnicity and geographic location. Therefore, we have evaluated the correlation between the AZFc subdeletion and fertility impairment in Siddi tribal group, who are descents from Africa and are genetically isolated population settled in Western Ghats of Karnataka. In an unexpected observation all our study subjects demonstrated the absence of deletion for *DAZ* gene cluster that encodes RNA-binding protein, which is exclusively expressed in the germ cells [48]. The deletion of each member of *DAZ* gene family (*DAZ1*, *DAZ2, DAZ3*, and *DAZ4*) is shown to have differential effect on male fertility and recent studies have reported various *DAZ* deletions in fertile men as well [49]. Thus, the requirement of *DAZ* gene for normal reproductive function and impact of deletion on fertility impairment across different ethnic groups remained as a major unsolved problem in human reproductive genetics.

Furthermore, screening of 12,000 patients and controls for the presence of gr/gr subdeletions demonstrate that this deletion occur more frequently in infertile men than fertile men, but prevalence may vary widely among fertile individuals [9]. Like *DAZ* deletion, the frequency of gr/gr deletion and its impact on fertility varies from one ethnic group to another. For example, 8% to 10% of deletions are observed in East Asian men with spermatogenic failure suggesting an increased risk of infertility [18,13], whereas, 1.4% of normozoospermic Chinese men with this deletion are normal for fertilization [50]. Previously as shown by Repping et al. [11], Lu et al. [51] and Yang et al. [52] these gr/gr deletions are fixed in haplogroups D2b and Q1, commonly observed in China and Japan and are anticipated not to hinder male infertility in patients as well as in controls [11,51,52]. Our current finding that the zero incidence of gr/gr deletion in Siddi tribal men additionally supports variation in deletion frequency observed across various ethnic groups. On the other hand, using sY1191 STS marker we report single b2/b3 deletion (0.50%) with no impact on reproductive function of an individual. In spite of continues attempts to correlate the b2/b3 deletion to semen morphological changes, if any, has failed as we could not retrieve semen sample from the subject. For that matter, due to strong mythological beliefs and other religion reasons none of the study subjects are volunteered to provide their semen sample. Thus, in the absence of semen morphological studies, our data is interpreted based on biochemical and molecular approaches. Nonetheless, our data is in consistency with previous studies in Northern European [49] and East Asian population, where occurrence of b2/b3 deletion is not associated with spermatogenic failure and infertility condition [12,53]. Indeed, various indirect studies partially agree with the proposition demonstrating that 80% of the b2/b3 deletions occurring in fertile Normozoospermic men belong to the deletion fixed haplogroup N [51]. The predisposition for the complete AZFc deletion has been identified in the b2/b3 deletion fixed haplogroup N and the clear link between b2/b3 deletions in the specific Y lineages and spermatogenic failure remains to be validated [12,51].

The observation that the low frequency occurrence of AZFc subdeletion, which has no impact on fertility in Siddi tribal men is in agreement with AZF deletion studies carried out in African population. Total of 49 infertile individuals were screened for Y chromosome microdeletions in AZFa, AZFb and AZFc regions [54]. Surprisingly, the absence of deletion in entire AZF regions among all the study subjects suggests that the observed infertility condition may possibly arise from deletion in other unknown subregions of Y chromosome or may be due to secondary sexual infections. Compared to non-African countries, 50% of African couples display secondary infertility due to sexual transmitted diseases (STD) and cultural practices such as polygamy, hinders the analysis of precise frequency of infertility or subfertility [55,56]. Though, Siddi tribes have an African ancestry, the practice of polygamy is not observed in this community and also, there are no reports of STD. Interestingly, the observed AZFc subdeletion is in very lower frequency (0.50%) matches to previous studies that accounts for 0% to 5.93% of b2/b3 deletion in fertile individuals with no association between these subdeletions and impairment in an individual’s fertility [17,19,51,57].

In conclusion, our study from South Indian tribal population establishes a negative co-relation between the AZFc subdeletion and its non association with fertility impairment. At this stage, we consider this as preliminary observation to definitively conclude the AZFc subdeletions in Siddi males are not as frequent as in other ethnic groups. Further, we hope to continue this investigation in a larger cohort to add on additional information on the overall frequency of Y chromosome AZFc subdeletions as well as to screen for additional AZFc STS markers to associate the genotypic variation to functional status of sperm. Finally, we suggest that examination of different ethnic groups in India for Y chromosome deletion may reveal novel deletion patterns that probably may have an adverse effect on fertilization and infertility rates. In future, this knowledge may be useful in diagnosing and developing new therapeutic approaches for diverse infertility conditions.

## Additional files

Additional file 1: Table S1. STS primer details: Information of the STS primer sequences that are employed in the current study for mapping the AZFc subdeletions. Additional file 2: Table S2. Age and marital status: Details of the study subject’s age group, marital status along with type of marriage, and different age group of children among married individuals, which shows their current reproductive status. Note that all the values are represented in percentage. Additional file 3: Table S3. Frequency of AZFc subdeletion mapping in Siddi tribal men.

## Abbreviations

AZFc: Azoospermic factor c
AZF: Azoospermic factor
FSH: Follicle stimulating hormone
LH: Leutinizing hormone
TAC: Total antioxidant capacity
SOD: Super oxide dismutase
STS: Sequence tagged site
MSY: Male-specifc Y
TCA: Trichloroaceticacid
BPY2: Basic protein Y
CDY: Chromodomain Y
DAZ: Deletion in azoospermia
GOLGAZLY: Golgi autoantigen golgin subfamily a2 like Y
CSPG4LY: Chondroitin sulphate proteoglycan 4 like Y
TTY: Testis transcript Y
EAA: European academy of andrology
EQMN: European molecular genetics quality network
ROS: Reactive oxygen species
IHEC: Institutional human ethical committee
OD: Optical density
STD: Sexually transmitted disease
