# Supplementary material for: Impact of Y chromosome AZFc subdeletion shows lower risk of fertility impairment in Siddi tribal men, Western Ghats, India
Source: Basic Clin Androl. 2015 Jan 22;25:1. doi: 10.1186/s12610-014-0017-5 (PMC4404687; doi:10.1186/s12610-014-0017-5)
Supplement: Additional file 1: Table S1. — STS primer details: Information of the STS primer sequences that are employed in the current study for mapping the AZFc subdeletions. [file 12610_2014_17_MOESM1_ESM.docx]

**Additional file 1 Table S1:** **STS primer details**: Information of the STS primer sequences that are employed in the current study for mapping the AZFc subdeletions

| **Sl. No.** | **STS marker** | **Deletion site in AZFc region** | **Forward**  **primer** | **Reverse**  **primer** | **PCR product size** | **Genbank accession number** |
| --- | --- | --- | --- | --- | --- | --- |
| 1 | sY254 | *DAZ* | GGGTGTTACCAGAAGGCAAA | GAACCGTATCTACCAAAGCAGC | 380bp | G38349 |
| 2 | sY255 | *DAZ* | GTTACAGGATTCGGCGTGAT | CTCGTCATGTGCAGCCAC | 123bp | G65827 |
| 3 | sY1291 | gr/gr | TAAAAGGCAGAACTGCCAGG | GGGAGAAAAGTTCTGCAACG | 527bp | G72340 |
| 4 | sY1191 | b1/b3 and b2/b3 | TCATTTGTGTCCTTCTCTTGGA | CTAAGCCAGGAACTTGCCAC | 453bp | G67168 |
| 5 | sY1197 | b1/b3 | CCAGACGTTCTACCCTTTCG | GAGCCGAGATCCAGTTACCA | 385bp | G73809 |
