# Supplementary material for: Impact of Y chromosome AZFc subdeletion shows lower risk of fertility impairment in Siddi tribal men, Western Ghats, India
Source: Basic Clin Androl. 2015 Jan 22;25:1. doi: 10.1186/s12610-014-0017-5 (PMC4404687; doi:10.1186/s12610-014-0017-5)
Supplement: Additional file 2: Table S2. — Age and marital status: Details of the study subject’s age group, marital status along with type of marriage, and different age group of children among married individuals, which shows their current reproductive status. Note that all the values are represented in percentage. [file 12610_2014_17_MOESM2_ESM.docx]

**Additional file 2: Table S2**: **Age and marital status**: Details of the study subject’s age group, marital status along with type of marriage, and different age group of children of married individuals which shows their current reproductive status. Note that all the values are represented in percentage.

| **Sl No** | **Age group** | **In %** | **Marital status** | **In %** | **Different age group children of married individuals** | **In %** |
| --- | --- | --- | --- | --- | --- | --- |
| 1 | 18-30 Yrs | 65% | Married | 52% | 1 Yr | 4.41% |
| 2 | 31-40 Yrs | 20% | Unmarried | 48% | 2 Yrs | 26.47% |
| 3 | 41 - 45 Yrs | 15% | Consanguineous | 44.24% | 3 Yrs | 41.92% |
| 4 | --- | --- | Non Consanguineous | 55.76% | 4 Yrs | 23.52% |
| 5 | --- | --- | --- | --- | 5 Yrs | 3.68% |
