# Supplementary material for: Impact of Y chromosome AZFc subdeletion shows lower risk of fertility impairment in Siddi tribal men, Western Ghats, India
Source: Basic Clin Androl. 2015 Jan 22;25:1. doi: 10.1186/s12610-014-0017-5 (PMC4404687; doi:10.1186/s12610-014-0017-5)
Supplement: Additional file 3: Table S3. — Frequency of AZFc subdeletion mapping in Siddi tribal men. [file 12610_2014_17_MOESM3_ESM.docx]

**Additional file 3: Table S3:** Frequency of AZFc subdeletion mapping in Siddi tribal men

| **Sl. No.** | **STS markers** | **Deletion loci in AZFc region** | **Occurrence of deletion**  **in married and unmarried group** | **Frequency in %**  **n=200**  **(104+96)** | **Condition** |
| --- | --- | --- | --- | --- | --- |
| 1 | sY254 | *DAZ* | Nil | 0% |  |
| 2 | sY255 | *DAZ* | Nil | 0% |  |
| 3 | sY1291 | gr/gr | Nil | 0% |  |
| 4 | sY1191 | b2/b3 | 1 | 0.96% | Married individual with 2 children |
| 5 | sY1197 | b1/b3 | Nil | 0% |  |
